# Association of the bovine aortic arch and bicuspid aortic valve with thoracic aortic disease

**DOI:** 10.1186/s12872-023-03095-0

**Published:** 2023-02-02

**Authors:** Jing Sun, Shuai Zhang, Hongxia Qi, Cheng Sun, Zhihui Hou, Xiaoqi Wang, Xiangyang Qian

**Affiliations:** 1 Department of Cardiovascular Surgery, National Center for Cardiovascular Diseases and Fuwai Hospital, Chinese Academy of Medical Sciences, Peking Union Medical College, No. 167, North Lishi Street, Xicheng District, Beijing, China; 2 Department of Cardiovascular Surgery, Yunnan Fuwai Cardiovascular Hospital, Kunming, China; 3 Department of Medical Imaging, Ultrasound Division, National Center for Cardiovascular Diseases and Fuwai Hospital, Chinese Academy of Medical Sciences, Peking Union Medical College, Beijing, China; 4 Department of Radiologic Imaging, National Center for Cardiovascular Diseases and Fuwai Hospital, Chinese Academy of Medical Sciences, Peking Union Medical College, Beijing, China

**Keywords:** Bicuspid aortic valve, Bovine aortic arch, Bicuspid aortopathy, Thoracic aortic disease

## Abstract

**Background:**

Both bicuspid aortic valve (BAV) and bovine aortic arch (BA) are considered as markers of thoracic aortic disease (TAD). But the association between them is not yet clear. This study aimed to explore the potential association of BAV and BA with TAD.

**Methods:**

The study involved 449 participants who underwent their first aortic valve replacement in Fuwai Hospital from June 2017 to March 2018. All patients underwent multidetector computed tomography and echocardiography before surgery. The clinical characteristics were recorded to analyze the association between BAV, BA, and TAD. The univariate and multivariate logistic regression analyses were applied to identify the risk factors for TAD.

**Results:**

BA accounted for 79.8% of the arch variants and was the most common aortic arch branching variant. BAV was present in 52.6% of the patients with BA and 38.1% of the patients with normal arch (NA). Among the 185 patients in the BAV subgroup, 50 had BA and 135 had NA. No significant differences were found in BAV anatomical phenotype, aortopathy phenotype, and valve function between BA and NA. The multivariate analysis showed that the presence of BAV and male sex were the risk predictors of TAD. BA was not a risk factor for TAD in either univariate or multivariate analysis.

**Conclusions:**

The proportion of BAV in patients with BA was significantly higher than that of NA, but the BAV phenotype and aortopathy were not related to BA. BAV was a risk factor for TAD, whereas BA was not associated with TAD.

## Introduction

Bovine aortic arch (BA) has been described as the most frequent variant of the aortic arch branching patterns in which the left common carotid artery originates from the innominate artery. Its prevalence has been reported to range from 7.2 to 35.16% according to the autopsy and radiologic studies [1, 2]. This incidence is lower in the Caucasian population and higher in the Black population [2]. The incidence in the Chinese population is 10.3% [3]. In the past, it was considered to be a “normal” anatomical variation without clinical significance. The presence of BA in patients with thoracic aortic disease (TAD) has, however, been extensively studied in recent years. Compared with the general population, patients with TAD have a higher prevalence of BA, which is associated with higher thoracic aortic growth rates [4–6], it has become a novel marker for TAD. Bicuspid aortic valve (BAV) is the most common congenital heart disease; approximately 45–50% of patients are associated with TAD, namely bicuspid aortopathy [7]. Therefore, BAV is also a well-known risk factor for TAD.

Studies have shown that patients harboring BAV or BA are more likely than the general population to develop TAD [4–7]. However, whether a connection exists between BAV and BA, which together lead to the high incidence of TAD, and what is the incidence of BA in patients with BAV are still unclear. Some studies involved in-depth exploration in the past. However, a few involved biomechanical characterization [8] and morphological descriptions [4]. Therefore, the present study was undertaken to investigate whether an association existed between BA and BAV and to explore their association with TAD.

## Patients and methods

### Patient population

**Fig. 1:**
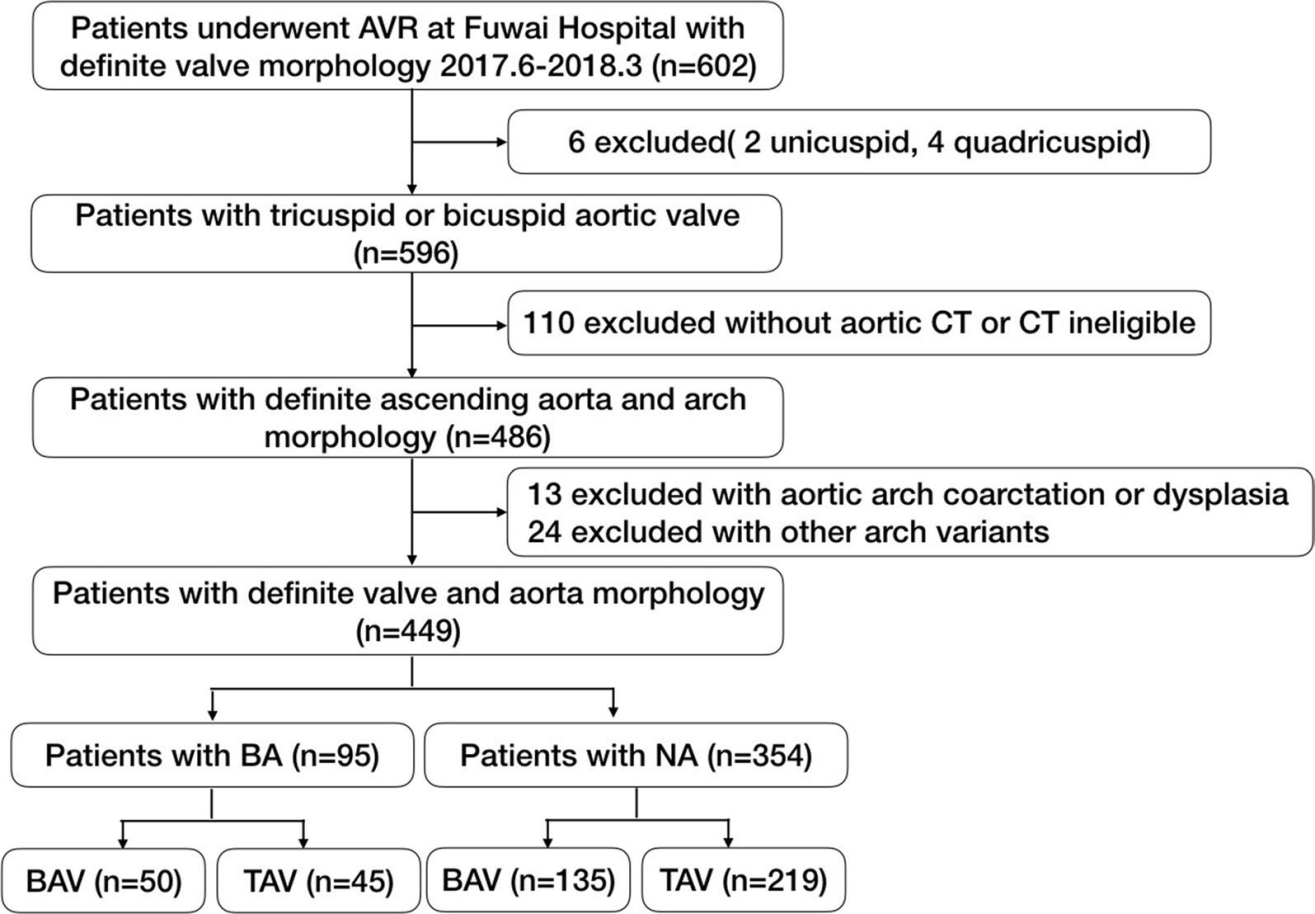
Flow chart of the study population. *AVR* Aortic valve replacement, *CT* computed tomography, *BA* Bovine aortic arch, *NA* Normal aortic arch, *BAV* Bicuspid aortic valve, *TAV* Tricuspid aortic valve.)

Form June 2017 to March 2018, 602 patients were scheduled to undergo their first aortic valve replacement (AVR) with intraoperative aortic valve morphology diagnosis at Fuwai Hospital in Beijing, China. The details of the screening process are depicted in Fig. 1. The aortic valve normally has three cusps or leaflets, although in 1–2% of the population it is found to congenitally have two leaflets. Excluding unicuspid (two patients) and quadricuspid (four patients) valves, only patients with bicuspid and tricuspid aortic valves were included in the present study. A total of 110 patients without aortic computed tomography (CT) examinations or substandard aortic CT images were excluded. Further, 13 patients with coarctation or dysplasia of the aortic arch were also excluded from the study. This study mainly focused on the BA. Therefore, the other 24 patients of aortic arch variants were also excluded: 21 patients had isolated left vertebral artery, 2 patients had BA combined with isolated left vertebral artery, and 1 patient had aberrant right subclavian artery. A total of 449 patients were finally enrolled, which included 95 patients with BA (50 with BAV and 45 with tricuspid aortic valve (TAV)) and 354 with normal arch (NA) (135 with BAV and 219 with TAV). The study protocol was approved by the Institutional Review Board of Fuwai Hospital, Peking Union Medical College (Beijing, China). Date and number of IRB approval: 7/2021, 2021-1494. The study was conducted following the World Medical Association Declaration of Helsinki code of ethics for research involving human participants. All patients provided written informed consent for their study participation.

### Definitions

During the AVR surgery, the chief surgeon assessed the valve morphology. The TAV is characterized by the presence of three commissures and three cusps. According to Sievers and Schmidtke [9], BAV morphology is defined by the number of raphes and the spatial position of those cusps. The categories included type 0 lateral, type 0 anterior–posterior, type 1 left coronary-right coronary cusp fusion, type 1 right coronary–noncoronary cusp fusion, and type 1 left coronary–noncoronary cusp fusion. Type 2 was excluded because it was considered to be a unicuspid aortic valve. We compared the prevalence of BAV in the BA and NA groups.

**Fig. 2:**
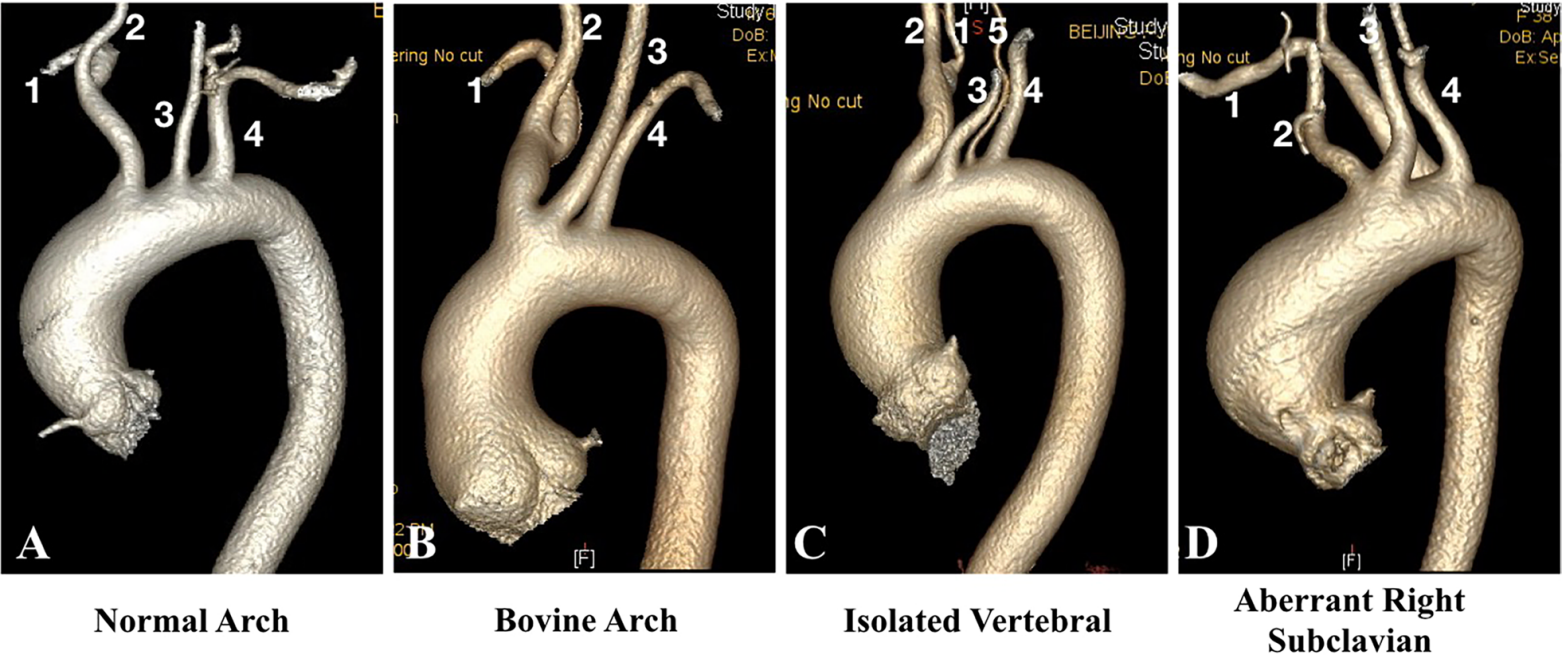
Aortic arch branching patterns: **A** normal aortic arch, **B** bovine aortic arch, **C** isolated left vertebral artery, **D** aberrant right subclavian artery. 1, Right subclavian artery; 2, right common carotid artery; 3, left common carotid artery; 4, left subclavian artery; 5, left vertebral artery

BA was determined by evaluating CT with three-dimensional reconstruction. NA was defined when the innominate artery, left common carotid artery, and left subclavian artery (LSA) originated, in turn, from the aortic arch. BA was defined when the left common carotid artery arose at a common origin or from a common trunk with the brachiocephalic artery. Two other common arch variants were also described in the present study. It is defined as a left vertebral artery arising directly from the aortic arch, either proximally or distally. In order to be defined as aberrant right subclavian artery, a fourth vessel must arise distal to the LSA and cross the posterior side of the mediastinum toward the upper right limb (Fig. 2).

TAD in the present study was defined as dilation of any segment of the thoracic aorta (aortic diameter ≥ 4.0 cm), including aortic sinus, ascending aorta, and aortic arch except the descending thoracic aorta. BAV aortopathy [7] was defined as dilatation of any or all segments of the proximal aorta from the aortic root to the aortic arch. It was divided into four phenotypes, namely normal, root, ascending, and arch phenotype, according to the dilation part. The functional state of the aortic valve was confirmed by echocardiography.

### Data collection

We used the electronic medical record system of the hospital to obtain information on demographic and clinical characteristics. The morphology of the aortic valves was assessed during the AVR surgery by the chief surgeon, and the excised valves were photographed in all included patients. Two observers (Cheng Sun and Ke Si) independently reviewed the surgery record and the photos of valves to determine the aortic valve phenotype. Two observers (Heng Zhang and Zhihui Hou) independently reviewed all CT images to determine the type of arch and measure the thoracic aortic diameter at seven levels (sinus of Valsalva, sinotubular junction, tubular portion of the ascending aorta, proximal to the innominate artery or common trunk in case of a BA, distal to the innominate artery or common trunk, proximal to the LSA, and distal to the LSA). In the case of a disagreement, a third observer (Jing Sun) reviewed the records and images to make the final decision.

### Statistical analysis

For descriptive statistical analysis, the continuous variables were normally distributed and were expressed as the mean ± standard deviation, and categorical variables as frequencies or percentages. The analysis of variance and the unpaired-sample Student *t* test were used to compare continuous variables among or between the groups. The categorical variables were analyzed using the chi-square and Fisher exact tests. The univariate and multivariate logistic regression analyses were performed to identify the risk factors for TAD. The results were displayed as odds ratio (OR) with 95% confidence interval (CI) and *P* value. SPSS version 19.0 software (SPSS, IL, USA) was used for the statistical analysis. A *P* value < 0.05 indicated a statistically significant difference.

## Results

### Characteristics of BA patients

Among the 449 patients in the present study, 95 (70 male and 25 female) were found to have a concomitant BA (a prevalence of 21.2%). If another 24 aortic arch branching variants were considered, BA accounted for 79.8% of the variants and was the most common aortic arch branching variant.

Table 1 shows the clinical profiles of the participants according to the BA and NA. No significant differences were found in terms of age, sex, body mass index (BMI), smoking history, diabetes, hypertension, and hyperlipidemia between BA and NA. BAV was present in 52.6% of the patients with BA and 38.1% with NA, which was a significant difference (*P* = 0.011). Although the diameter of the ascending aorta was larger in the BA group than in the NA group, the difference was not statistically significant.

**Table 1:** Demographic characteristics and risk factors in the BA and NA groups

Variables	All (*n* = 449)	BA (*n* = 95)	NA (*n* = 354)	*P* Value
Age (year)	55.1 ± 12.7	54.5 ± 12.5	55.3 ± 12.8	0.588
Male sex	330 (73.5)	70 (73.7)	260 (73.4)	0.963
BMI	24.3 ± 3.6	24.3 ± 4.1	24.3 ± 3.5	0.974
Smoking	324 (72.2)	70 (73.7)	254 (71.8)	0.575
Diabetes mellitus	38 (8.5)	10 (10.5)	28 (7.9)	0.416
Hypertension	199 (44.3)	39 (41.1)	160 (45.2)	0.506
Hyperlipidemia	229 (51.0)	47 (49.5)	182 (51.4)	0.662
BAV	185 (41.2)	50 (52.6)	135 (38.1)	0.011
Ascending aorta (mm)	47.1 ± 12.0	48.3 ± 13.2	46.8 ± 11.6	0.256
NYHA functional class				0.519
I	57(12.7)	12(12.6)	45(12.7)	
II	223(49.7)	50(52.6)	173(48.9)	
III	163(36.3)	32(33.7)	131(37.0)	
IV	6(1.3)	1(1.1)	5(1.4)	
TAD	324 (72.2)	70 (73.7)	254 (71.8)	0.709

Values are presented as *n* (column %) or mean ± standard deviation

*BA* Bovine arch, *NA* Normal arch, *BMI* Body mass index, *BAV* Bicuspid aortic valve, *TAD* Thoracic aortic disease

#### Characteristics of BAV patients

After clarifying the incidence of BAV in patients with BA, we further explored the association of BA with BAV phenotype, valve function, and BAV aortopathy. The detailed data are shown in Table 2. Among the 185 patients in the BAV subgroup, 50 had BA and 135 had NA. No significant differences were found in the age, sex, BMI, smoking history, diabetes, hypertension, diabetes mellitus, hyperlipidemia, cardiac function, and TAD between BA and NA in the BAV subgroup. Also, no significant differences were observed in BAV anatomical phenotype, aortopathy phenotype, and valve function between the two groups.

**Table 2:** Demographic characteristics and risk factors in patients with BAV

Variables	All (*n* = 185)	NA (*n* = 135)	BA(*n* = 50)	*P* value
Age (year)	53.8 ± 12.6	53.1 ± 12.9	55.7 ± 11.8	0.23
Male sex	137 (74.1)	101 (74.8)	36 (72)	0.70
BMI	24.5 ± 3.6	24.4 ± 3.5	24.6 ± 4.2	0.78
Smoking	143 (77.3)	103 (76.3)	40 (80)	0.60
Diabetes mellitus	18 (9.7)	12 (8.9)	6 (12)	0.53
Hypertension	65 (35.1)	45 (33.3)	20 (40)	0.40
Hyperlipidemia	95 (51.4)	72 (53.3)	23 (46)	0.38
NYHA functional III/IV	55 (29.7)	40 (29.6)	15 (30)	0.96
TAD	155 (83.8)	114 (84.4)	41 (81)	0.69
Sievers’ classification				0.15^*^
0-lat	56 (30.3)	46 (34.1)	10 (20)	
0-ap	15 (8.1)	11 (8.1)	4 (8)	
1-L-R	85 (45.9)	57 (42.2)	28 (56)	
1-R-N	18 (9.7)	15 (11.1)	3 (6)	
1-N-L	11 (5.9)	6 (4.4)	5 (10)	
Aortic valve function				0.47^*^
AS	85 (45.9)	65 (48.1)	20 (40)	
AI	63 (34.1)	46 (34.1)	17 (34)	
AS/AI	15 (8.1)	11 (8.1)	4 (8)	
Non-AS/AI	22 (11.9)	13 (9.6)	9 (18)	
Aortopathy				0.25^*^
Normal	34 (18.4)	24 (17.8)	10 (20)	
Root	14 (7.6)	13 (9.6)	1 (2)	
Ascending	92 (49.7)	67 (49.6)	25 (50)	
Arch	45 (24.3)	31 (23)	14 (28)	

Values are presented as *n* (%) or mean ± standard deviation

*BA* Bovine arch, *NA* Normal arch, *BMI* Body mass index, *BAV* Bicuspid aortic valve, *TAD* Thoracic aortic disease, *NYHA* New York Heart Association Functional Classification, The codification for the Sievers’ classification (Type 0 for value with no raphe; Type 1 for valve with one raphe); *ap* Anterior–posterior, *lat* Lateral, *L* Left coronary sinus, *R* Right coronary sinus, *N* Non-coronary sinus, *AS* Aortic stenosis, *AI* Aortic insufficiency

*Overall *P* value of χ^2^ analysis of Sievers’ classification, aortic valve function, and aortopathy

### Risk factors for TAD

We included seven baseline variables, BAV, and BA to analyze the risk factors of TAD (Table 3). The univariate analysis showed that BAV and male sex were the risk factors for TAD. The multivariate analysis reconfirmed the presence of BAV (OR 2.345; 95% CI 1.46–3.767; *P* < 0.001) and male sex (OR 1.886; 95% CI 1.159–3.069; *P* = 0.011) as the predictors of TAD. BA was not a risk factor for TAD in either univariate or multivariate analysis.

**Table 3:** Risk factors for TAD

	Univariate		Multivariate	
Variable	OR (95% CI)	*P* value	OR (95% CI)	*P* value
BA	1.102 (0.661–1.839)	0.709	0.952 (0.555–1.632)	0.857
BAV	2.105 (1.350–3.283)	0.001	2.345 (1.460–3.767)	< 0.001

The multivariate model adjusted age, sex, BMI, tobacco use, hypertension, diabetes mellitus, hyperlipidemia, BAV, and BA to analyze the risk factors of TAD

*BA* Bovine arch, *BAV* Bicuspid aortic valve

## Discussion

The present study found that the proportion of BAV in patients with BA was significantly higher than that of the NA, but the BAV phenotype and aortopathy were not related to BA. BAV was a risk factor for dilation of the ascending aorta, but no significant correlation was found between BA and TAD.

In the past, BA, an aortic arch branching pattern that is most common, was considered a normal anatomical variant with no hemodynamic or pathological significance. BA has gained more interest among cardiac and vascular surgeons in the last 10 years. Based on autopsy and radiologic studies, its prevalence ranges from 7.2 to 35.16% [1, 2]. In the present study, the BA prevalence was 21.2%, which was higher than the 10.3% incidence of BA in the Chinese population reported by Lei Wang and co-workers [3]. The difference in the incidence of BA might be related to the difference in the study population. The present study population comprised all patients with aortic valve disease or aneurysm undergoing surgical treatment, while Lei Wang’s study population was a broader CT examination population. In other studies on TAD, BA also had a higher incidence. In the study by Julia Dumfarth and co-workers [10], the incidence of BA in type A aortic dissection was 15.6%. Spyridon N. Mylonas and co-workers [11] found that the incidence of BA in type B aortic dissection was 17.7%. The study by Matthew Hornick and co-workers [6] found that the incidence of BA in TAD was as high as 26.3%. Pamela A. Moorehead and co-workers [12] found a prevalence of BA of 31% in their study cohort, and BA prevalence showed an upward trend in patients with thoracic aortic dissection (42.3% vs 30.8%; *P* = 0.28). However, studies based on fetal populations found that the incidence of BA was only 4.8% [13]. The incidence of BA varied greatly among different study populations. Therefore, a large number of cardiac and vascular surgeons speculated that BA might have certain clinical significance.

In recent years, BA has been shown to be a marker of TAD based on more stringent case–control studies [4–6, 14]. The team of Doctor John A. Elefteriades from Yale-New Haven Hospital has done a lot of work in this area. They compared two groups of patients with dilated and nondilated aortic disease, and found that atypical aortic arch branching variants, especially BA, were the risk factors for TAD [4, 6]. As a result of a similar study from the University of California, San Francisco, CA, USA, it was reported by Malone et al. [5] that chest CT and magnetic resonance imaging studies were reviewed retrospectively in 191 patients with dilated thoracic aortas and 391 controls. Compared with controls, patients with BA had a higher incidence of dilated thoracic aorta, but only those over 70 years old (31.9 vs 16.0%; *P* = 0.016). In the present study, the incidence of a TAD was not statistically different between patients with BA and NA (73.7 vs 71.8%; *P* = 0.709). In our further TAD risk factor analysis, we found that BA was not a risk factor for TAD, using either univariate or multivariate analysis.

At present, whether a BA is associated with aortopathy is not certain. More studies, including hemodynamic studies, histological studies, and prospective cohort studies (in the absence of aortic dilatation), need to be performed to determine whether BA was a risk factor for TAD. T. Pham and co-workers [8] investigated and compared the mechanical and microstructural properties of aortic tissues from patients with ascending aortic aneurysms with and without concomitant BAV or BA. The study found the BAV samples were stiffer and thinnest with less elastin compared with both simple ascending aortic aneurysm and BA samples. The BA samples were similar to the simple ascending aortic aneurysm samples in terms of both mechanical and failure properties. This study found no histomechanical evidence for BA as a risk factor for ascending aortic aneurysms. Four-dimensional flow MRI was used by Sherene Shalhub and co-workers [15] to investigate the association between type B aortic dissection and variant arch anatomy and hemodynamic mechanisms. Compared with the NA, BA showed significant flow acceleration in the descending aorta inner curve, creating elevated regional wall shear stress. The blood flow in the ascending aorta and aortic arch of BA was indistinguishable from that in the NA. This study found no hemodynamic evidence for BA as a risk factor for ascending aortic aneurysms.

In the present study, although BA was not a risk factor for TAD, BAV was a risk factor for TAD. The proportion of BAV in patients with BA was significantly higher than that in patients with NA (52.6 vs 38.1%; *P* = 0.011). Few studies explored whether an association existed between BA and BAV. Matthew Hornick and co-workers [6] found that patients with BA and non-BA patients with TAD who had BAV were 26.7% and 25.3% respectively, which was not a significant difference (*P* = 0.68). Julia Dumfarth and co-workers [4] found that patients with aortic aneurysms and aortic arch anomalies had a higher rate of BAV (40.8% vs 30.6%; *P* = 0.042). 73.7% of the members of their research cohort had the BA pattern as the most common anomalous branch pattern. The proportion of BAV in BA in the present study was significantly higher than that in the study by Matthew Hornick [6] and Julia Dumfarth [4]. Possibly, there is a biological link between aortic valve morphology and arch morphology (either genetically or embryologically). Therefore, we further explored the association between BAV phenotype, aortopathy, and valve function with BA, but unfortunately, we did not find a link between them.

## Limitations

The limitations of this study included the retrospective and single-institution study design. All patients undergoing heart valve surgery were enrolled, and the incidence of TAD was high, leading to some selection bias. Hence, the results of the study could not be generalized to a wider population. Additionally, a small sample size limited the ability to provide meaningful analysis of statistical significance in some cases.

## Conclusions

Our analysis revealed a prevalence of BA of 21.2%, the most common aortic arch branching variant. The proportion of BAV in patients with BA was significantly higher than that of TAV, but the BAV phenotype and aortopathy were not related to BA. BA was not a risk factor, whereas BAV and male sex were risk factors, for ascending aortic dilatation.

## Data Availability

The datasets used and/or analysed during the current study are available from the corresponding author on reasonable request.

## Abbreviations

BAV: Bicuspid aortic valve
BA: Bovine aortic arch
TAD: Thoracic aortic disease
NA: Normal arch
AVR: Aortic valve replacement
CT: Computed tomography
LSA: Left subclavian artery
OR: Odds ratio
CI: Confidence interval
NYHA: New York Heart Association Functional Classification
ap: Anterior–posterior
lat: Lateral
L: Left coronary sinus
R: Right coronary sinus
N: Non-coronary sinus

## References

[1] Ahn SS, Chen SW, Miller TJ, Chen JF (2014). What is the true incidence of anomalous bovine left common carotid artery configuration?. Ann Vasc Surg.

[2] Clerici G, Giulietti E, Babucci G, Chaoui R (2018). Bovine aortic arch: clinical significance and hemodynamic evaluation. J Matern Fetal Neonatal Med.

[3] Wang L, Zhang J, Xin S (2016). Morphologic features of the aortic arch and its branches in the adult Chinese population. J Vasc Surg.

[4] Dumfarth J, Chou AS, Ziganshin BA, Bhandari R, Peterss S, Tranquilli M, Mojibian H, Fang H, Rizzo JA, Elefteriades JA (2015). Atypical aortic arch branching variants: a novel marker for thoracic aortic disease. J Thorac Cardiovasc Surg.

[5] Malone CD, Urbania TH, Crook SE, Hope MD (2012). Bovine aortic arch: a novel association with thoracic aortic dilation. Clin Radiol.

[6] Hornick M, Moomiaie R, Mojibian H, Ziganshin B, Almuwaqqat Z, Lee ES, Rizzo JA, Tranquilli M, Elefteriades JA (2012). 'Bovine' aortic arch: a marker for thoracic aortic disease. Cardiology.

[7] Verma S, Siu SC (2014). Aortic dilatation in patients with bicuspid aortic valve. N Engl J Med.

[8] Pham T, Martin C, Elefteriades J, Sun W (2013). Biomechanical characterization of ascending aortic aneurysm with concomitant bicuspid aortic valve and bovine aortic arch. Acta Biomater.

[9] Sievers HH, Schmidtke C (2007). A classification system for the bicuspid aortic valve from 304 surgical specimens. J Thorac Cardiovasc Surg.

[10] Dumfarth J, Peterss S, Kofler M, Plaikner M, Ziganshin BA, Schachner T, Tranquilli M, Grimm M, Elefteriades JA (2017). In DeBakey type I aortic dissection, bovine aortic arch is associated with arch tears and stroke. Ann Thorac Surg.

[11] Mylonas SN, Barkans A, Ante M, Wippermann J, Bockler D, Brunkwall JS (2018). Prevalence of bovine aortic arch variant in patients with aortic dissection and its implications in the outcome of patients with acute type B aortic dissection. Eur J Vasc Endovasc Surg.

[12] Moorehead PA, Kim AH, Miller CP, Kashyap TV, Kendrick DE, Kashyap VS (2016). Prevalence of bovine aortic arch configuration in adult patients with and without thoracic aortic pathology. Ann Vasc Surg.

[13] Goldsher YW, Salem Y, Weisz B, Achiron R, Jacobson JM, Gindes L (2020). Bovine aortic arch: prevalence in human fetuses. J Clin Ultrasound.

[14] Ikeno Y, Koide Y, Matsueda T, Yamanaka K, Inoue T, Ishihara S, Nakayama S, Tanaka H, Sugimoto K, Okita Y (2019). Anatomical variations of aortic arch vessels in Japanese patients with aortic arch disease. Gen Thorac Cardiovasc Surg.

[15] Shalhub S, Schafer M, Hatsukami TS, Sweet MP, Reynolds JJ, Bolster FA, Shin SH, Reece TB, Singh N, Starnes BW (2018). Association of variant arch anatomy with type B aortic dissection and hemodynamic mechanisms. J Vasc Surg.

